# Dynamic Behavior of Mass Sensor Based on Switchable Dual-Mode Composite Strips

**DOI:** 10.3390/s26113342

**Published:** 2026-05-25

**Authors:** Yuekai Xu, Haohao Bi

**Affiliations:** School of Science, Qingdao University of Technology, Qingdao 266520, China; yuekaixu00aza@126.com

**Keywords:** film–substrate composite strips, dual-mode, three-dimensional buckled, mass perturbations, mass sensor

## Abstract

Micro- and nanoscale mass sensing is crucial for applications such as molecular detection and wearable monitoring. However, the observation of mass perturbations in flexible composite structures requires systematic theoretical evaluation. This study develops a dual-mode vibration-based mass-sensing model based on a film–substrate composite strip. By releasing and re-stretching pre-strain in the soft substrate, the ribbon can reversibly switch between a two-dimensional flat configuration (Mode 1) and a three-dimensional buckled configuration (Mode 2), leading to distinct dynamic responses. Under a finite-deformation Euler–Bernoulli beam assumption, displacement fields and kinematic relations are formulated for both configurations. An energy-based approach is employed to decompose the total energy into stretching and bending contributions, while an added-mass block is incorporated into the kinetic energy as a lumped mass. The governing equations of motion are derived using the Lagrange equations and the Hamiltonian function. Based on these results, the influence of the added mass on displacement signatures is examined, and the mode-dependent observability in the flat versus buckled states is compared, providing an analytical basis for mass sensor evaluation.

## 1. Introduction

In recent years, flexible electronic materials such as graphene [1,2,3] and polyimide [4] have shown great potential in applications like wearable monitoring and soft-body interactions [5,6,7,8,9] due to their lightweight, bendable/stretchable nature, ability to fit complex curved surfaces, and stable functionality under dynamic deformations. Small-scale mass changes, such as those caused by gas molecules, biological molecules, and viruses, are often manifested as differences in the period and amplitude of the response through changes in structural dynamic parameters. Meanwhile, composite materials with strips covered by thin films exhibit higher response sensitivity and observability of small mass loads under vibration readout. Therefore, vibration-based mass sensors are of significant importance in flexible electronics, micro-material detection, and structural condition monitoring: they can transform tiny, hard-to-measure masses into precise dynamic signals that can be read, providing a feasible path for the development of highly sensitive detection and mass sensor technologies. In addition, recent studies have shown that resonance-frequency shifts and mode-dependent dynamic responses are effective readout signals for micro/nanoscale mass sensing. Frequency-shift modeling has been used to identify the attached mass and its position in small-scale beams [10], and sequential frequency-shift measurements have been applied to particle identification in micro-plates [11]. Moreover, broader studies on micromachined mechanical resonant sensors have summarized the development of resonant mechanisms, structural designs, and sensing applications [12]. These works provide a useful background for the present dual-mode vibration-based mass-sensing model. In addition, nonlinear vibration-based mass sensing has been reported to provide additional response features compared with conventional linear frequency-shift models. Kang et al. [13] showed that nonlinear oscillation changes the resonance-response characteristics of CNT resonators and affects the mass-induced frequency-shift behavior. This suggests that retaining nonlinear dynamic effects can provide richer sensing information than purely linear approximations, which motivates the nonlinear framework adopted in the present work.

To increase the amount of information obtained and enhance the identifiability of parameters, both domestic and international studies have proposed using switchable vibration modes generated by the same structure in different configurations to acquire more dynamic responses. By leveraging the release and re-stretching of pre-strain in the underlying soft substrate [4,14,15], this structure can undergo deterministic buckling [14,16,17,18,19,20,21,22] under mechanical guidance, enabling reversible switching between two-dimensional flat and three-dimensional buckled states, thus yielding two significantly different dynamic responses. Based on this approach, Wang et al. used an adjustable three-dimensional structure to achieve the parallel determination of the film’s modulus and density [23]; Zhao et al. applied these two vibration configurations to the mass and modulus characterization of small-scale biological systems, such as cells or organoids [24].

With the continuous advancement of research on two-dimensional materials such as boron nitride [25] and molybdenum disulfide [26], traditional two-dimensional structures face certain limitations that do not exist in three-dimensional structures. Therefore, by extending two-dimensional structures into three-dimensional wrinkled and buckled configurations, it is possible not only to preserve some of the excellent material properties of the original two-dimensional films but also to exhibit new material and structural characteristics. In flexible electronic structures, a typical approach is to combine rigid films with flexible substrates and induce buckling of the film structure by releasing pre-strain, enabling the device to maintain material advantages while achieving adjustable three-dimensional geometric forms. In studies of these wrinkled/buckled structures, Lee et al. investigated flexible sensors based on film/substrate structures with a wrinkled configuration [7]; Li et al. explored a nanomechanical mass sensor based on the multi-directional vibration of buckled strips [27]; and Park et al. introduced wrinkles into carbon nanotube films, enhancing elasticity and pressure sensitivity [28]. In addition, previous experimental studies have demonstrated soft three-dimensional vibratory platforms assembled by compressive buckling. Nan et al. developed flexible PI/Au/PI composite vibratory structures on an elastomeric substrate and measured their multimodal amplitude and frequency responses using Lorentz-force actuation and optical detection. These results support the feasibility of fabricating buckled film–substrate vibratory structures for sensing applications [14].

This article presents a mass sensor model based on dual-mode strips, which combines the vibrational response characteristics of both flat and buckled states. The model aims to improve the sensitivity and identifiability of mass/gravity sensing by exploiting the significant differences between these two states. Specifically, the composite ribbon structure consists of a film and substrate, with rapid switching between the flat state (Mode 1) and the buckled state (Mode 2) achieved through the release and re-stretching of the pre-strained substrate. The model employs finite-deformation Euler–Bernoulli beam theory to describe the geometric features and displacement fields of both the flat and buckled states. The system’s dynamic equations are derived using the Lagrange equations and the Hamiltonian function, allowing the dynamic response of the system to be obtained. The second part of this article establishes the dual-mode composite strip model, the third and fourth parts present and analyze the results obtained from the model, and the fifth part draws the final conclusions.

## 2. Dual-Mode Composite Strips Model

**Figure 1:**
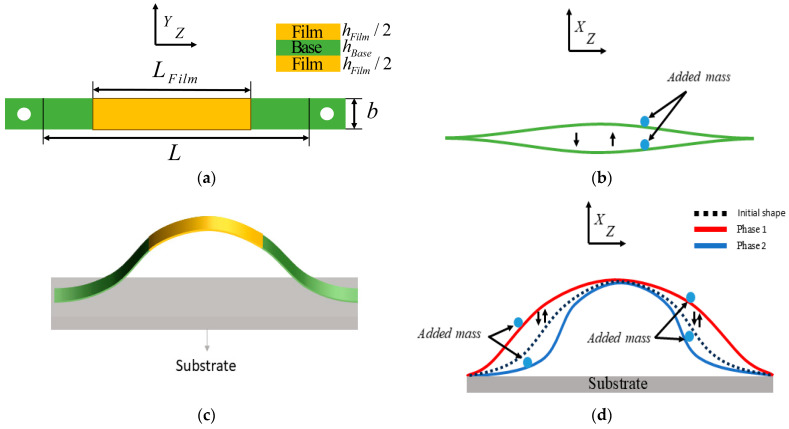
Two vibration modes of the strip: (**a**) a schematic diagram of a planar strip covered with a thin film; (**b**) the vibration mode of Mode 1 with an additional mass block; (**c**) the wavy strip with a thin film in Mode 2; and (**d**) the vibration mode of Mode 2, with additional mass blocks at different locations. Phase 1 and Phase 2 correspond to the maximum amplitude during the vibration process.

This section establishes a finite beam model for the feasibility analysis of a dual-mode mass sensor, as shown in Figure 1. The model places the film in the region $-L_{\mathrm{Film}}/2\leq Z\leq L_{\mathrm{Film}}/2$, with the two ends of the strip attached to a pre-strained elastic substrate. Releasing the pre-strain causes the strip to bend into an arch shape, thus generating two different modes: Mode 1 (flat state) and Mode 2 (buckled state) [4]. Consider a strip of length L, width b, and thickness $h_{Base}$ and investigate the effect of an additional mass block $m_{k}$ at $z=z_{k}$. Assume the strip thickness is much smaller than its width and length, i.e., $h_{Base}\ll b$ and $h_{Base}\ll L$, thus introducing the finite-strain Euler beam theory [29,30]. Here, the X direction is assumed to be perpendicular to the strip outwards, the Y direction along the strip width, and the Z direction along the strip axis.

### 2.1. Theoretical Derivation

Let $u_{3}$ be the axial displacement of the strip, $u_{2}$ be the width displacement of the strip, and $u_{1}$ be the out-of-plane displacement of the strip. Because the considered film–substrate strip has a slender geometry and its internal stiffness is much greater than its external bending stiffness, the width-direction displacement is ignored in the following derivation [23]. Let $\Delta a\left(t\right)$ be the vibration displacement.

To characterize the geometric nonlinearity under finite deformation, curvature κ and stretch ratio λ are introduced. Here, κ represents the geometric strength of the neutral axis bending [4,31], and λ represents the degree of deformation of the material after being subjected to tensile force [27].

In order to degenerate to the conventional linear beam theory under small deflection limit and to retain the nonlinear coupling terms caused by geometric compatibility under buckled state and large deflection vibration, this paper adopts geometric nonlinear approximations of κ and λ, derived from [32,33].

(1) $$\kappa =\frac{\partial^{2}u_{1}}{\partial z^{2}}$$

(2) $$\lambda =1+\frac{\partial u_{3}}{\partial z}+\frac{1}{2}{\left(\frac{\partial u_{1}}{\partial z}\right)}^{2}$$

The total mechanical energy of the vibrating belt is divided into kinetic energy (defined as $T(\Delta \dot{a})$) and potential energy (defined as $W_{s}$), among which the potential energy of the vibrating belt is composed of contributions from the film and the strip, and can be divided into two parts: tensile strain energy (defined as $W_{m}$) and bending energy (defined as $W_{b}$). This energy is contributed by both the base and film components. The tensile strain energy $W_{m}$ (Equation (3)) corresponds to the tensile energy caused by the elongation of the neutral axis, and the bending energy $W_{b}$ (Equation (4)) corresponds to the bending energy caused by thin film bending. Since the strip length is L, it is integrated over $\left[-L/2,L/2\right]$, and the film length is $L_{Film}$, so it is integrated over $\left[-L_{\mathrm{Film}}/2,L_{\mathrm{Film}}/2\right]$. $E_{Base}$ is the base strip modulus, $E_{Film}$ is the film modulus, $h_{Base}$ is the base strip thickness, and $h_{Film}$ is the film thickness.

(3) $$\begin{array}{cc} W_{m} & =\frac{1}{2}\int_{-L/2}^{L/2}E_{Base}bh_{Base}{\left(\lambda -1\right)}^{2}dz+\frac{1}{2}\int_{-L_{Film}/2}^{L_{Film}/2}E_{Film}bh_{Film}{\left(\lambda -1\right)}^{2}dz\\ & =\frac{1}{2}\int_{-L/2}^{L/2}E_{Base}bh_{Base}{\left[\frac{\partial u_{3}}{\partial z}+\frac{1}{2}{\left(\frac{\partial u_{1}}{\partial z}\right)}^{2}\right]}^{2}dz+\frac{1}{2}\int_{-L_{Film}/2}^{L_{Film}/2}E_{Film}bh_{Film}{\left[\frac{\partial u_{3}}{\partial z}+\frac{1}{2}{\left(\frac{\partial u_{1}}{\partial z}\right)}^{2}\right]}^{2}dz \end{array}$$

(4) $$\begin{array}{cc} W_{b} & =\frac{1}{24}\int_{-L/2}^{L/2}E_{Base}bh_{Base}^{3}\kappa^{2}dz+\frac{1}{8}\int_{-L_{Film}/2}^{L_{Film}/2}E_{Film}bh_{Film}h_{Base}^{2}\kappa^{2}dz\\ & =\frac{1}{24}\int_{-L/2}^{L/2}E_{Base}bh_{Base}^{3}{\left(\frac{\partial^{2}u_{1}}{\partial z^{2}}\right)}^{2}dz+\frac{1}{8}\int_{-L_{Film}/2}^{L_{Film}/2}E_{Film}bh_{Film}h_{Base}^{2}{\left(\frac{\partial^{2}u_{1}}{\partial z^{2}}\right)}^{2}dz \end{array}$$

The total strain energy is denoted as $W_{s}$, which can be regarded as a potential energy term in a conservative system without external forces.

(5) $$\begin{array}{cc} W_{s} & =W_{b}+W_{m}\\ & =\frac{E_{\mathrm{Base}}bh_{\mathrm{Base}}^{3}}{L^{3}}\left(k_{0}+k_{1}\Delta a+k_{2}\Delta a^{2}+k_{3}\Delta a^{4}\right)\\ & +\frac{E_{\mathrm{Film}}bh_{\mathrm{Film}}h_{\mathrm{Base}}^{2}}{L^{3}}\left(k_{0,\mathrm{Film}}+k_{1,\mathrm{Film}}\Delta a+k_{2,\mathrm{Film}}\Delta a^{2}+k_{3,\mathrm{Film}}\Delta a^{4}\right)\\ & =K_{0}+K_{1}\Delta a+K_{2}\Delta a^{2}+K_{3}\Delta a^{4}+K_{0,\mathrm{Film}}+K_{1,\mathrm{Film}}\Delta a+K_{2,\mathrm{Film}}\Delta a^{2}+K_{3,\mathrm{Film}}\Delta a^{4} \end{array}$$

(6) $$K_{i}=\frac{E_{\mathrm{Base}}bh_{\mathrm{Base}}^{3}}{L^{3}}k_{i}\;(i=0,1,2,3)$$

(7) $$K_{i,Film}=\frac{E_{\mathrm{Base}}bh_{\mathrm{Base}}^{2}h_{Film}}{L^{3}}k_{i,Film}\;(i=0,1,2,3)$$

The kinetic energy is composed of the beam itself and the added-mass block, where the kinetic energy of the beam itself is obtained by the superposition of the translational kinetic energy of the base layer and the thin-film layer. The added mass is assumed to be much smaller than the total mass of the composite strip; therefore, it is modeled as a concentrated point mass at $z=z_{k}$. Here, $\rho_{Base}$ is the base density, $\rho_{Film}$ is the thin-film density, m is the mass of the base strip, $m_{Film}$ is the mass of the thin film, and $\Delta \dot{a}$ is the first derivative of the vibration displacement $\Delta a\left(t\right)$ with respect to time.

(8) $$\begin{array}{ll} T_{1}(\Delta \dot{a}) & =\frac{1}{2}\int_{-L/2}^{L/2}\rho_{\mathrm{Base}}bh_{\mathrm{Base}}\left[{\left(\frac{\partial u_{1}}{\partial t}\right)}^{2}+{\left(\frac{\partial u_{3}}{\partial t}\right)}^{2}\right]\mathrm{dz}\\ & +\frac{1}{2}\int_{-LFilm/2}^{LFilm/2}\rho_{\mathrm{Film}}bh_{\mathrm{Film}}\left[{\left(\frac{\partial u_{1}}{\partial t}\right)}^{2}+{\left(\frac{\partial u_{3}}{\partial t}\right)}^{2}\right]\mathrm{dz}\\ & =(m\rho_{\mathrm{Base}}bh_{\mathrm{Base}}L+m_{Film}\rho_{Film}bh_{Film}L)\Delta {\dot{a}}^{2}\\ & =\frac{1}{2}M_{n}\Delta {\dot{a}}^{2} \end{array}$$

The added mass $m_{k}$ is regarded as a concentrated mass located at $z=z_{k}$, and its kinetic energy $T_{2}$ can be represented by the Dirac function $\delta (z-z_{k})$ in the length integral to achieve the equivalent representation of the point mass [27], thus obtaining Equation (7).

(9) $$T_{2}(\Delta \dot{a})=\frac{1}{2}\sum_{k=1}^{N}m_{k}\delta (Z-Z_{k})\left[{\left(\frac{\partial u_{1}}{\partial t}\right)}^{2}+{\left(\frac{\partial u_{3}}{\partial t}\right)}^{2}\right]=\frac{1}{2}M_{m}\Delta {\dot{a}}^{2}$$

The Dirac function $\delta (z-z_{k})$ takes the value of

(10) $$\delta (z-z_{k})=\left\{\begin{array}{c} 1,z=z_{k},\\ 0,z\neq z_{k}. \end{array}\right.$$

The Dirac delta function is introduced to equivalently represent the concentrated added mass located at $z=z_{k}$ in the continuous beam model. Since the added mass moves with the local velocity at its attachment position, its kinetic energy contribution should be determined by the velocity at that point. In the integral formulation, the Dirac delta function extracts the local velocity term at $z=z_{k}$, thereby incorporating the kinetic energy of the point mass into the continuous energy expression.

Total kinetic energy is

(11) $$T(\Delta \dot{a})=T_{1}(\Delta \dot{a})+T_{2}(\Delta \dot{a})=\frac{1}{2}M\Delta {\dot{a}}^{2},\;M=M_{n}+M_{m}$$

Under this modeling, the mass block enters the system only through kinetic energy, without directly changing $W_{s}$.

### 2.2. Mode 1

To achieve Mode 1, the substrate is stretched to make the strip into a two-dimensional flat state, thereby realizing the planar vibration mode of Mode 1, and the displacement function comes from [23]. The strip displacement for Mode 1 is

(12) $$u_{1}(t,z)=\Delta a(t)\left[1+\cos\left(\frac{2\pi z}{L}\right)\right]$$

(13) $$u_{3}(t,z)=0$$

By substituting the displacement fields into Equations (1)–(11), the strain energy and kinetic energy can be reduced to polynomial forms in terms of the generalized coordinate (The derivation process is presented in Appendix A.1). The resulting coefficients m, $m_{Film}$, $k_{i}$ and $k_{i,Film}$ $\left(i=0,1,2,3\right)$ are obtained through direct integration, and their explicit expressions are provided in Appendix A.3.

### 2.3. Mode 2

To achieve Mode 2, the two ends of the strip are selectively bonded to a pre-stretched elastic substrate. After the pre-strain is released, the strip buckles and forms an arched structure, thus realizing the wavy strip vibration mode of Mode 2, and the displacement function comes from [23]. The strip displacement for Mode 2 is

(14) $$u_{1}(t,z)=A_{\left(0\right)}\left[\cos\left(\frac{2\pi z}{L}\right)+1\right]+\Delta a(t)\left[1-\cos\left(\frac{4\pi z}{L}\right)\right]$$

(15) $$u_{3}(t,z)=\frac{\pi A_{\left(0\right)}^{2}}{4L}\sin\left(\frac{4\pi z}{L}\right)-\varepsilon_{compre}z+\Delta a(t)\frac{\pi A_{\left(0\right)}}{3L}\left[6\sin\left(\frac{2\pi z}{L}\right)-2\sin\left(\frac{6\pi z}{L}\right)\right]$$

where $A_{\left(0\right)}=\frac{L}{\pi }\sqrt{\varepsilon_{compre}-\frac{\pi^{2}h_{Base}^{2}}{3L^{2}}}$ is the static buckling deflection amplitude and $\varepsilon_{compre}$ is the compressive strain, which is used to reflect the equivalent compression in the axial direction after the pre-strain is released [23]. Unlike the flat state, the axial displacement $u_{3}$ in the buckled state cannot be simply ignored, and the coefficients can be obtained in the same way (The derivation process is presented in Appendix A.2). The resulting coefficient m, $m_{Film}$, $k_{i}$ and $k_{i,Film}$$\left(i=0,1,2,3\right)$ are obtained through direct integration, and their explicit expressions are provided in Appendix A.4.

### 2.4. Lagrange Equations of Motion

(16) $$\frac{\partial (T-W_{s})}{\partial \Delta a}-\frac{d}{dt}\left[\frac{\partial (T-W_{s})}{\partial \Delta \dot{a}}\right]=0$$

(17) $$M\Delta \ddot{a}+(K_{1}+K_{1,\mathrm{Film}})+2(K_{2}+K_{2,\mathrm{Film}})\Delta a+4(K_{3}+K_{3,\mathrm{Film}})\Delta a^{3}=0$$

where $\Delta \ddot{a}$ is the second derivative of the vibration displacement $\Delta a\left(t\right)$ with respect to time.

To obtain the amplitude-dependent nonlinear natural frequency, the first-order harmonic balance method is applied to Equation (17). Since Equation (17) describes free vibration without damping or external harmonic excitation, the following result represents the nonlinear frequency-amplitude backbone relation. Since the displacement appears periodic with respect to time from the image, let us assume

(18) $$\Delta a(t)=A_{0}+A_{1}\cos\left(\omega t\right)$$

Substituting into Equation (17) and then using the harmonic balance method, we can obtain the following equation (the derivation process is presented in Appendix A.5).

(19) $$\omega^{2}=\frac{2\left(K_{2}+K_{2,Film}\right)+12\left(K_{3}+K_{3,Film}\right)A_{0}^{2}+3\left(K_{3}+K_{3,Film}\right)A_{1}^{2}}{M}$$

### 2.5. Hamiltonian Function Representation

Define the generalized momentum conjugate with Δa as follows:

(20) $$p=\frac{\partial \left(T-W_{s}\right)}{\partial \Delta \dot{a}}=M\Delta \dot{a}$$

The Hamiltonian function Equation (21) is obtained, which is a conserved quantity under the condition of no damping and no external force.

(21) $$H\left(\Delta a,p\right)=p\Delta \dot{a}-\left(T-W_{s}\right)=\frac{p^{2}}{2M}+W_{s}\left(\Delta a\right)$$

## 3. Dynamic Behavior and Model Verification

In this section, the initial geometry and material properties of the buckled strips are set to L=10 μm, $L_{\mathrm{Film}}$=6 μm, b=5 nm, $h_{B\mathrm{ase}}$=1.5 μm, $h_{\mathrm{Film}}$=0.34 nm, $E_{B\mathrm{ase}}$=2.5 GPa, $E_{\mathrm{Film}}$=1.0 TPa, $\rho_{Base}$$=1420\;\mathrm{kg}/m^{3}$, $\rho_{Film}$$=2250\;\mathrm{kg}/m^{3}$, $\varepsilon_{compre}$=0.25 and the material coefficients are from [23,27]. In addition, γ represents linear density, γL represents the quality of the strip, and the added-mass block is denoted by $m_{mass}$. Since the added mass considered in this study is much smaller than the mass of the strip $\left(m_{mass}=0.001\gamma L-0.01\gamma L\right)$, its influence on the structural configuration is neglected.

### 3.1. Finite Element Analysis

To demonstrate whether the analytical displacement function used in this paper can reasonably reflect the basic vibration modes under the two configurations, this paper introduces linear finite element mode shape results for comparison. This comparison mainly focuses on the spatial distribution characteristics of the mode shape along the strip length, observing the end constraint performance, peak position, curve symmetry, and the participation of different displacement components in the vibration.

**Figure 2:**
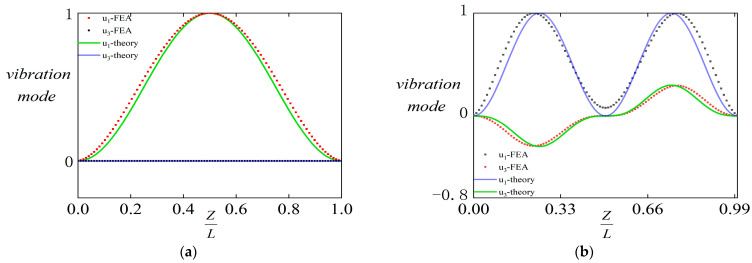
Finite element analysis. (**a**) Mode 1. (**b**) Mode 2.

As shown in Figure 2a, for Mode 1, the finite element results show that the structural deformation is mainly reflected in the transverse displacement component $u_{1}$, while the axial displacement component $u_{3}$ is relatively small and can be approximately ignored. This is consistent with the displacement assumption of treating Mode 1 as transverse bending vibration under a flat reference configuration.

As shown in Figure 2b, for Mode 2, since the structure is in a buckled configuration, the vibration response is no longer described by a single transverse component. The finite element results and the analytical mode shape maintain consistency in the main variation trends, indicating that the displacement function selected in this paper can accurately describe the main deformation characteristics under both the flat and buckled states.

Based on this, it is reasonable to use this analytical mode shape to establish the energy expression, derive the Lagrange equations, and construct the Hamiltonian function.

### 3.2. Graph Analysis of Hamiltonian Function and Potential Function

#### 3.2.1. Mode 1

**Figure 3:**
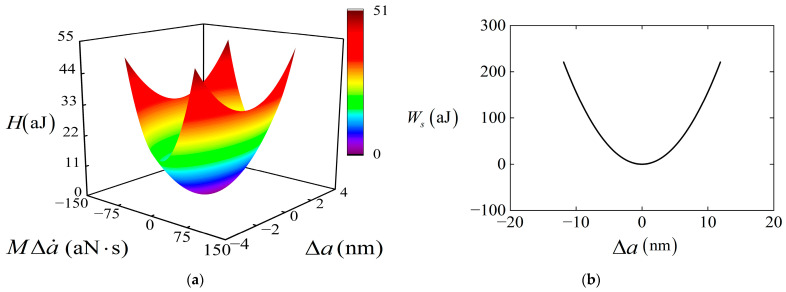
Mode 1 without mass blocks. (**a**) Hamiltonian function, (**b**) potential function. The curve in (**b**) represents the potential-energy curve $W_{s}\left(\Delta a\right)=H\left(\Delta a,0\right)$.

The Hamiltonian function for Mode 1 is $H\left(\Delta a,p\right)=p^{2}/2M+W_{s}\left(\Delta a\right)$, where $p=M\Delta \dot{a}$. Figure 3a shows the energy surface of a typical single-potential-well structure. From the functional form, we can see that the energy along the p direction increases twice with the generalized momentum and is symmetrical about p=0, exhibiting a parabolic opening shape. The shape of Δa along the generalized coordinate direction is determined by $W_{S}\left(\Delta a\right)$, thus forming the single-potential-well energy surface. The lowest point of the energy surface is located at p=0 and $dW_{s}/d\Delta a=0$ (i.e., when $\Delta a=K_{1}+K_{1,Film}=0$), corresponding to the elliptical stable equilibrium point in the phase plane. Therefore, in the undamped case, the system phase space trajectory is along the contour line of H=E (E is a constant), forming a closed loop around the potential well near the equilibrium point, reflecting stable periodic oscillation.

In Figure 3b, the potential function satisfies $W_{s}\left(\Delta a\right)=H\left(\Delta a,0\right)$, which is the projection of the Hamiltonian function onto section p=0. The figure shows a single potential well with the opening facing upwards, and the only minimum value in the figure is exactly the same as the lowest point in Figure 3a.

In summary, the Hamiltonian energy surface and potential function image of Mode 1 together indicate that the system exhibits a convex single-potential-well energy basin under the current parameters, with a unique stable equilibrium point and a family of closed isoenergetic lines.

#### 3.2.2. Mode 2

**Figure 4:**
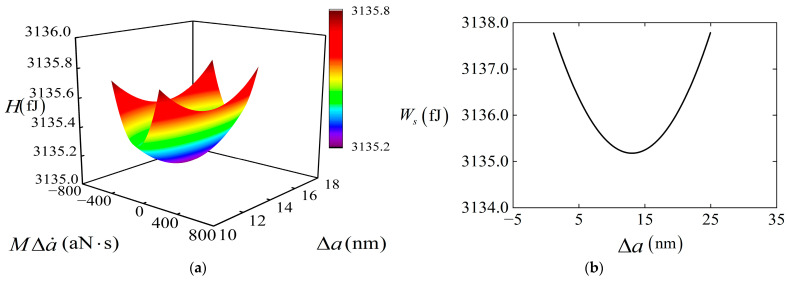
Mode 2 without mass blocks. (**a**) Hamiltonian function, and (**b**) potential function. The curve in (**b**) represents the potential-energy curve $W_{s}\left(\Delta a\right)=H\left(\Delta a,0\right)$.

The Hamiltonian function for Mode 2 is $H\left(\Delta a,p\right)=p^{2}/2M+W_{s}\left(\Delta a\right)$, where $p=M\Delta \dot{a}$. Since the function form remains essentially unchanged, the shapes of Figure 4a,b are still single-potential-well energy basins. However, unlike Mode 1, the lowest point is significantly shifted in the Δa direction because $dW_{s}/d\Delta a=K_{1}+K_{1,\mathrm{Film}}$ is not zero, thus shifting along Δa. Therefore, this also reflects that Mode 2 forms a closed loop around the single potential well near the equilibrium point and undergoes stable periodic oscillations.

## 4. Added-Mass Sensing Response

### 4.1. The Mass Blocks Are Located in Different Positions

#### 4.1.1. Mode 1

Since the mass block only affects the kinetic energy term, its location will only affect the $p^{2}/2M$ term of the Hamiltonian function. Since $T_{2}$ represents the kinetic energy of the strip and the film itself, it will only affect $T_{2}$, as shown in Equation (7). Furthermore, because the mass block is considered to have the same mass, the influence of different locations is determined by the square of the $\left[{\left(\partial u_{1}/\partial t\right)}^{2}+{\left(\partial u_{3}/\partial t\right)}^{2}\right]$ velocity at location $z=z_{k}$, thus affecting the equivalent mass coefficient $M_{m}$ and consequently the Hamiltonian function graph. However, since the difference $M_{m}$ only affects the magnitude of M, the shape of the function graph itself will not change; only the size of the quadratic function opening along the p direction will change.

**Figure 5:**
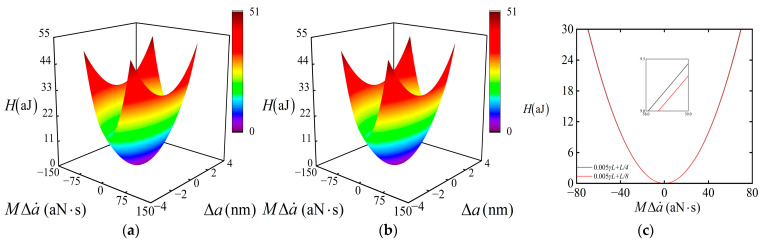
The Hamiltonian function for Mode 1 with mass blocks of mass 0.005γL placed at positions (**a**) z=L/4 and (**b**) z=L/8. (**c**) Hamiltonian section at the zero generalized displacement $\left(\Delta a=0\right)$.

Since Mode 1 is a two-dimensional flat state with $u_{3}=0$, the influence on displacement is only reflected through $u_{1}$, and it still exhibits regular periodic oscillations at different positions (Figure 5). Two differences are observed in $u_{1}$: firstly, phase and period differences, meaning the peaks appear at different rates within the same time window, with some curves showing a shift in peak value and a slower oscillation rhythm compared to the state without a mass block (i.e., none); secondly, amplitude differences, meaning the peak and trough heights change with placement, but the waveform remains smooth and exhibits regular periodic sinusoidal oscillations without abrupt changes in shape. This indicates that the nature of periodic oscillation within the stable potential well remains unchanged.

**Figure 6:**
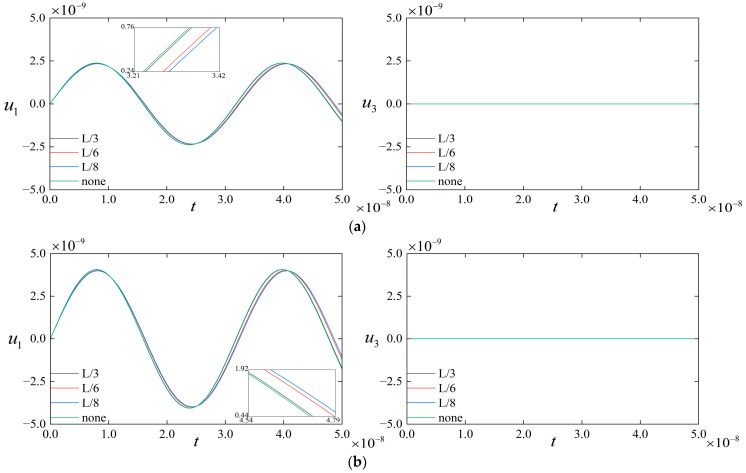
The time history diagram of the displacement at positions z=L/4 and z=L/8 when a mass block of mass 0.005γL is placed at different positions in Mode 1: (**a**) at z=L/4 and (**b**) at z=L/8.

The phenomenon in Figure 6 can be explained by the composition of the kinetic energy term. According to Equations (6)–(9), the mass block only introduces an additional kinetic energy contribution through Equation (7), and the point value characteristics of $\delta \left(z-z_{k}\right)$ determine that it is only related to the velocity of $z=z_{k}$. Both figures show that $u_{1}$ produces a stable and discernible difference, indicating that Mode 1 has observable sensitivity to different mass block positions.

#### 4.1.2. Mode 2

**Figure 7:**
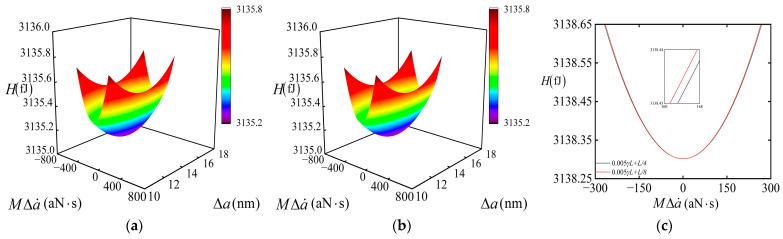
The Hamiltonian function for Mode 2 with mass blocks of mass 0.005γL placed at positions (**a**) z=L/4 and (**b**) z=L/8. (**c**) Hamiltonian section at the zero generalized displacement $\left(\Delta a=0\right)$.

Similarly, since the mass block only affects the kinetic energy term, its location in different positions will only affect the $p^{2}/2M$ term of the Hamiltonian function. At the same time, as shown in Figure 7, it will not affect the stable potential well structure itself. Moreover, for the Hamiltonian function, there will be a significant difference in the size of the quadratic function opening in the p direction, thus showing strong sensitivity to different positions of the mass block.

**Figure 8:**
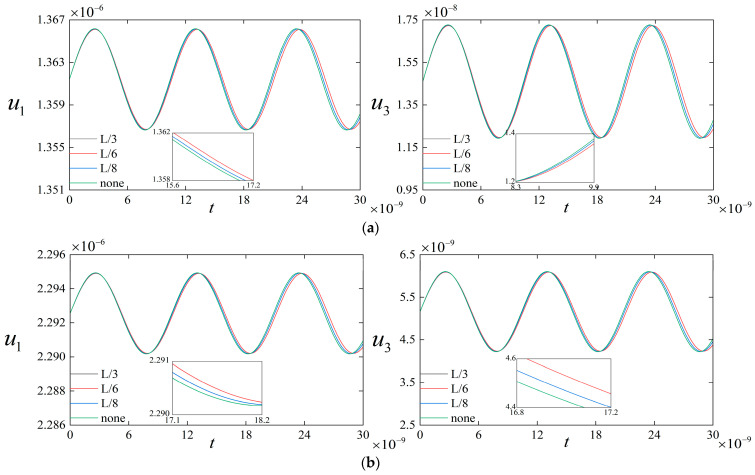
The time history diagram of the displacement at positions z=L/4 and z=L/8 when a mass block of mass 0.005γL is placed at different positions in Mode 2: (**a**) at z=L/4 and (**b**) at z=L/8.

Since Mode 2 is a three-dimensional buckled state, displacements $u_{1}$ and $u_{3}$ can both exhibit regular periodic vibrations. Only their periods and amplitudes show significant differences (Figure 8), indicating that the added-mass block does not cause the periodic vibration of the stable potential well itself, but it can produce significant differences in period and amplitude to reflect the different effects brought about by the different positions of the mass block.

Meanwhile, since Mode 2 has two variables, $u_{1}$ and $u_{3}$, which can reflect the differences at different positions and the changes are synchronous, it reflects that the two components in Mode 2 are driven by the same generalized coordinate, and the out-of-plane component is no longer negligible. This provides a basis for improving the observability of mass block position recognition by using multi-channel output.

Unlike Mode 1, Mode 2 affects not only the displacement $u_{1}$ but also both displacement $u_{1}$ and displacement $u_{3}$, making the observation results more reliable and helping to determine the location of the mass block.

#### 4.1.3. Amplitude and Frequency Response

Let $A_{u_{1}}$ be the amplitude of $u_{1}$, $A_{u_{3}}$ be the amplitude of $u_{3}$, and f be the frequency.

**Figure 9:**
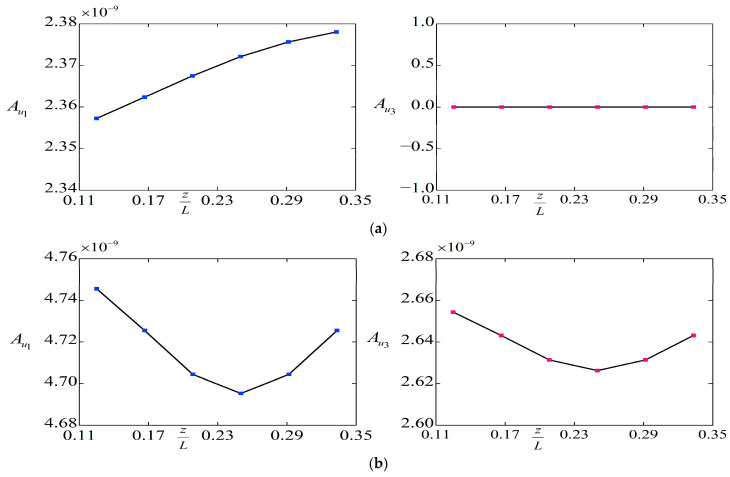
Effect of the added-mass location on the displacement amplitudes of the two vibration modes. The added mass is fixed at 0.005γL, and the amplitudes are evaluated at the observation point z=L/4 for different mass locations. The curves represent the variations of $A_{u_{1}}$ and $A_{u_{3}}$ with the normalized added-mass location $\frac{z}{L}$. (**a**) Mode 1. (**b**) Mode 2.

Figure 9 shows the effect of the added-mass location on the displacement amplitude. When the added mass is fixed at 0.005γL, Mode 1 mainly exhibits an $A_{u_{1}}$-dominated response, while $A_{u_{3}}$ remains nearly zero. In contrast, both $A_{u_{1}}$ and $A_{u_{3}}$ in Mode 2 vary with the mass location, showing a decreasing-then-increasing trend with relatively small values near the middle position.

**Figure 10:**
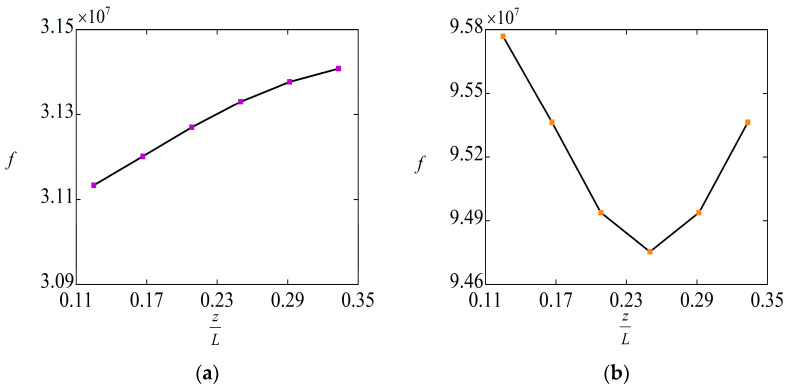
Effect of the added-mass location on the vibration frequency of the two vibration modes. The added mass is fixed at 0.005γL, and the frequency is extracted from the response at the observation point z=L/4 as the mass location varies along the strip. The curves represent the variation of the vibration frequency f with the normalized added-mass location $\frac{z}{L}$. (**a**) Mode 1. (**b**) Mode 2.

Figure 10 shows the corresponding frequency response. As the mass location changes, the frequency of Mode 1 gradually increases, whereas the frequency of Mode 2 first decreases and then increases. This indicates that the same added mass produces different frequency-shift patterns in the flat and buckled configurations.

The complementarity of the two modes is reflected in their different responses to the same added mass at different locations. In Mode 1, changing the mass location mainly leads to approximately monotonic variations in amplitude and frequency, but the available information is mainly concentrated in one dominant displacement component. In Mode 2, the same position variation produces another type of response feature: the amplitude response is non-monotonic, and the frequency response also first decreases and then increases. Therefore, Mode 2 does not simply repeat the information obtained from Mode 1 but provides additional position-dependent response features through the buckled configuration. When different mass locations produce similar responses in one mode, the other mode can provide supplementary information through its different amplitude and frequency variation patterns.

The advantage of the dual-mode configuration lies in the increased amount of observable response information. The flat configuration provides a relatively direct position-dependent response, while the buckled configuration offers additional amplitude features and a different frequency variation pattern. Therefore, displacement amplitude and vibration frequency can be used as measurable readout quantities, and combining these responses in the two modes helps improve the distinguishability of different added-mass locations.

#### 4.1.4. Nonlinear Harmonic Response Analysis

**Figure 11:**
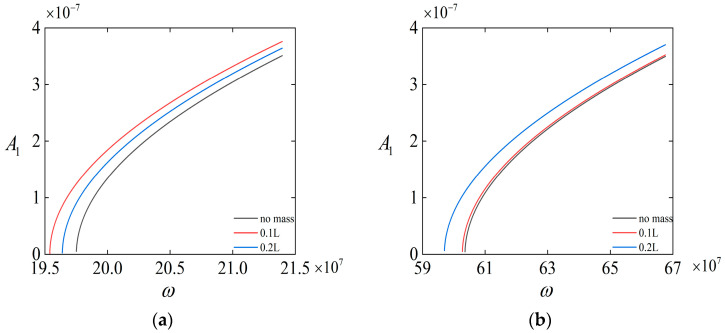
Nonlinear amplitude–frequency response curves of the two vibration modes under different added-mass locations. (**a**) Mode 1. (**b**) Mode 2.

Figure 11 shows the nonlinear amplitude–frequency response curves of the two vibration modes under different added-mass locations. In both Mode 1 and Mode 2, the vibration frequency increases with the amplitude, indicating a nonlinear hardening-type response. This confirms that the retained higher-order nonlinear terms affect the amplitude-dependent frequency response of the system.

For Mode 1, different added-mass locations lead to distinguishable shifts of the nonlinear response curves. Compared with the no-mass case, the curves with added mass generally shift toward lower frequencies, which is consistent with the increase in equivalent inertia caused by the attached mass. In addition, the curves corresponding to different mass locations do not overlap, indicating that the nonlinear frequency response is sensitive to the added-mass location.

For Mode 2, the nonlinear amplitude–frequency curves also vary with the added-mass location, but the curve distribution is different from that of Mode 1. This indicates that the buckled configuration provides an additional mode-dependent nonlinear response feature. Therefore, the nonlinear harmonic response further supports the feasibility of using the dual-mode configuration to distinguish different added-mass locations.

### 4.2. Mass Blocks of Different Sizes

#### 4.2.1. Mode 1

**Figure 12:**
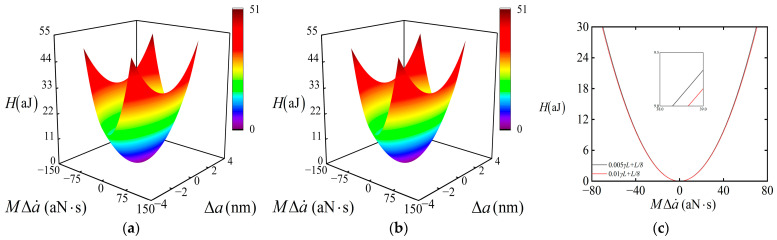
Hamiltonian function of Mode 1 with mass blocks of mass 0.01γL and 0.005γL placed at positions: (**a**) 0.01γL (**b**) 0.005γL. (**c**) Hamiltonian section at the zero generalized displacement $\left(\Delta a=0\right)$.

Since the mass block only affects the kinetic energy term, it actually only affects $T_{2}$. When placing mass blocks of different sizes at the same position, Equation (7) can be obtained as an equivalent scaling of the equivalent mass coefficient $M_{m}$. In Figure 12, when the mass changes from 0.001γL to 0.01γL, $T_{2}$ increases, which leads to an increase in M, making the parabola along the p direction flatter. Therefore, the change in the Hamiltonian function image is consistent with the change in the formula, and the image remains stable, representing a single-potential-well energy surface.

**Figure 13:**
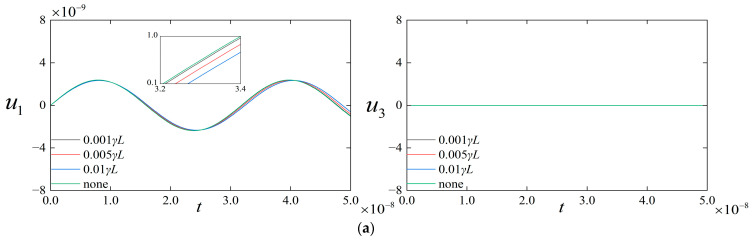

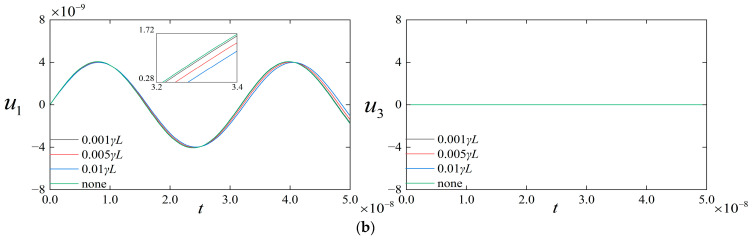
The time history diagram of the displacement at z=L/4 and z=L/8 when different mass blocks of different masses are placed at position z=L/8 in Mode 1: (**a**) at z=L/4 and (**b**) at z=L/8.

As shown in Figure 13, the displacement time histories given in Figure 13a,b for Mode 1 both exhibit stable periodic vibrations. First, $u_{3}$ is always zero in both subfigures, indicating that the response of Mode 1 can be regarded as a single-component motion dominated by the axial displacement $u_{3}$. The added mass does not affect the out-of-plane displacement in this mode, so the main information for subsequent comparisons focuses on the change of $u_{1}$.

Furthermore, a comparison of Figure 13a,b shows that the trends of mass change on the two observation points are consistent. Moreover, the comparison in Figure 13 shows that under different masses, significant differences can be found in the displacement versus time history graph, which are reflected in the differences in amplitude and period. Therefore, this provides identification features for mass identification.

#### 4.2.2. Mode 2

**Figure 14:**
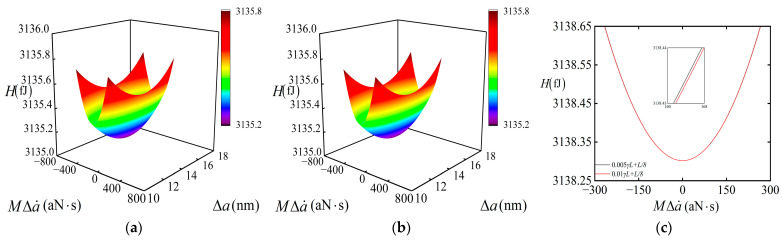
Hamiltonian function of Mode 2 with mass blocks of mass 0.01γL and 0.005γL placed at positions (**a**) 0.01γL (**b**) 0.005γL. (**c**) Hamiltonian section at the zero generalized displacement $\left(\Delta a=0\right)$.

Similarly, since the mass block only affects the kinetic energy term, its location in different positions will only affect the $p^{2}/2M$ term of the Hamiltonian function. At the same time, as shown in Figure 14, it will not affect the stable potential well structure itself. Moreover, for the Hamiltonian function, there will be a significant difference in the size of the quadratic function opening in the p direction, thus making it highly sensitive to the identification of different mass sizes.

**Figure 15:**
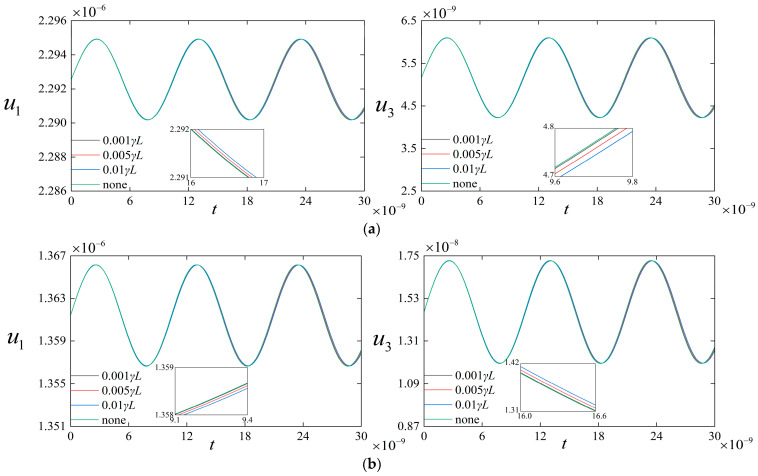
The time history diagram of the displacement at z=L/4 and z=L/8 when different mass blocks of different masses are placed at position z=L/8 in Mode 2: (**a**) at z=L/4 and (**b**) at z=L/8.

Similarly, since Mode 2 is a three-dimensional buckled state, displacements $u_{1}$ and $u_{3}$ can both exhibit regular periodic vibrations. Only their periods and amplitudes show significant differences (Figure 15), indicating that the added-mass block does not cause the periodic vibration of the stable potential well itself, but it can produce significant differences in period and amplitude to reflect the different effects brought about by the different masses of the mass block.

Meanwhile, since Mode 2 has two variables, displacement $u_{1}$ and displacement $u_{3}$, which can reflect the differences produced by different masses, and the changes show a synchronous trend, it reflects that the two components in Mode 2 are driven by the same generalized coordinate, and the out-of-plane component is no longer negligible. This provides a basis for improving the observability of mass size identification by using multi-channel output.

#### 4.2.3. Amplitude and Frequency Response

Let $A_{u_{1}}$ be the amplitude of $u_{1}$, $A_{u_{3}}$ be the amplitude of $u_{3}$, and f be the frequency.

**Figure 16:**
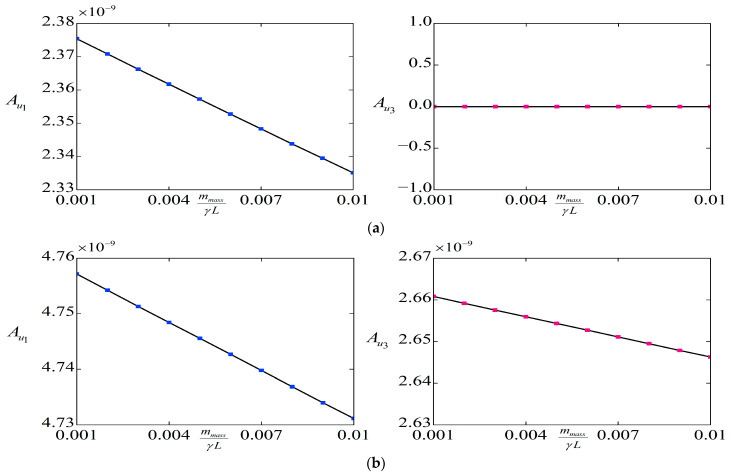
Effect of the added-mass magnitude on the displacement amplitudes of the two vibration modes. The added mass is fixed at z=L/8, and the displacement amplitudes are evaluated at the observation point z=L/4 for different added-mass values. The curves represent the variations of $A_{u_{1}}$ and $A_{u_{3}}$ with the normalized added-mass location $\frac{m_{mass}}{\gamma L}$. (**a**) Mode 1. (**b**) Mode 2.

Figure 16 shows the effect of the added-mass magnitude on the displacement amplitude when the mass location is fixed at z=L/8. As $m_{mass}$ increases, Mode 1 mainly exhibits a gradually decreasing $A_{u_{1}}$ amplitude response, while $A_{u_{3}}$ remains nearly zero. In contrast, both $A_{u_{1}}$ and $A_{u_{3}}$ in Mode 2 decrease with the increase in added mass, indicating that the buckled configuration can provide additional amplitude-response information under the same mass-location condition.

**Figure 17:**
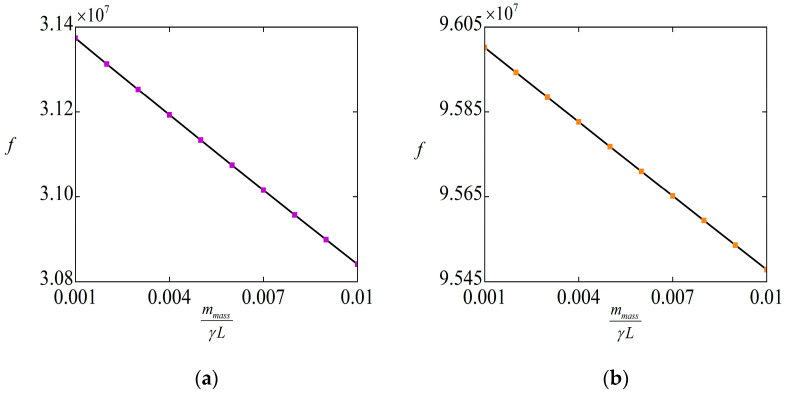
Effect of the added-mass magnitude on the vibration frequency. The mass location is fixed at z=L/8, and the frequency is extracted from the displacement response at z=L/4. The curves represent the variation of the vibration frequency f with the normalized added-mass magnitude $\frac{m_{mass}}{\gamma L}$. (**a**) Mode 1. (**b**) Mode 2.

Figure 17 shows the corresponding frequency response. When the mass location is fixed and $m_{mass}$ increases, the frequencies of both Mode 1 and Mode 2 decrease. This trend is consistent with the increase in equivalent inertia caused by the added mass. However, their specific frequency values are significantly different. Therefore, the frequency response can be used to reflect the variation in added-mass magnitude.

The complementarity of the two modes is reflected in their different responses to the same mass variation at a fixed location. In Mode 1, the increase in $m_{mass}$ mainly causes a monotonic decrease in the $A_{u_{1}}$ amplitude response and the corresponding frequency response. Therefore, Mode 1 provides a relatively direct mass-dependent signal, but the available information is mainly limited to a single amplitude response. In Mode 2, the same increase in $m_{mass}$ leads to coupled amplitude changes in two displacement components, and its frequency response also decreases at a different rate of variation. This indicates that Mode 2 provides additional mass-related features that are absent in Mode 1. When the mass-variation information in one mode is insufficient for reliable identification, the other mode can provide supplementary information through different amplitude and frequency response levels.

The advantage of the dual-mode configuration lies in the increased amount of observable information for mass identification. Under a fixed attachment position, both amplitude and frequency can be used as measurable readout quantities, and the combination of these responses in the flat and buckled configurations helps improve the distinguishability of different added-mass magnitudes.

#### 4.2.4. Nonlinear Harmonic Response Analysis

**Figure 18:**
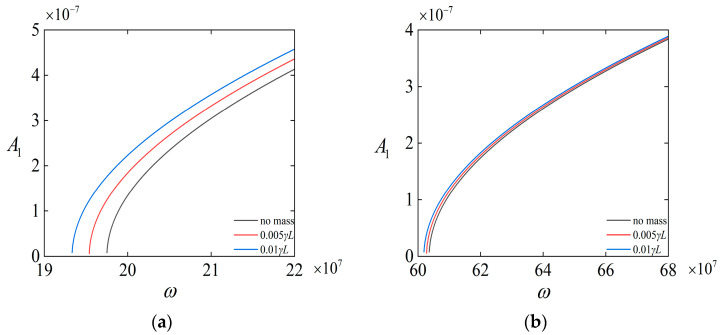
Nonlinear amplitude–frequency response curves of the two vibration modes under different added-mass magnitudes. (**a**) Mode 1. (**b**) Mode 2.

Figure 18 shows the nonlinear amplitude–frequency response curves of the two vibration modes under different added-mass magnitudes. In both Mode 1 and Mode 2, the vibration frequency increases with the vibration amplitude, indicating a nonlinear hardening-type response. This result shows that the nonlinear terms retained in the present model lead to an amplitude-dependent frequency response.

For Mode 1, increasing the added mass causes a clear shift of the nonlinear response curve toward lower frequencies. This is consistent with the increase in equivalent inertia caused by the added mass. Therefore, the nonlinear amplitude–frequency curve of Mode 1 is sensitive to the added-mass magnitude.

For Mode 2, the curves corresponding to different added-mass magnitudes are closer to each other but still show distinguishable differences. This indicates that the buckled configuration also reflects the effect of mass variation through the nonlinear amplitude–frequency response, although its sensitivity pattern is different from that of Mode 1. Therefore, the nonlinear harmonic response further confirms that different added-mass magnitudes can be reflected through the amplitude-dependent frequency characteristics of the two modes.

## 5. Conclusions

This article discusses the feasibility of using a dual-mode mass sensor, establishes an energy-type analytical model of the covered thin-film strip in both flat and buckled states, treats the additional mass as a point mass and includes it in the kinetic energy term, derives the Lagrange equations of motion, constructs the Hamiltonian function, and draws conclusions through graphs of the Hamiltonian function and displacement.

1. Both modes exhibit a single-potential-well stable system, with the Hamiltonian energy surface being a single-potential-well basin. The lowest point corresponds to an elliptical stable point in the phase plane, indicating that the system undergoes stable periodic motion without damping or external force.
2. When the mass block is placed at different locations, the opening of the Hamiltonian energy surface in the momentum direction differs, manifesting as stable differences in phase, period, and amplitude in the displacement time history. Both modes exhibit observable sensitivity to position changes. Mode 2, in particular, shows differences not only in a single displacement component but also simultaneously in two displacement components, which can improve the reliability of position determination.
3. Placing mass blocks of different masses at a fixed position causes a systematic change in the parabolic opening of the Hamiltonian function in the momentum direction; the corresponding time-domain response exhibits stable and distinguishable differences in the period and amplitude. In Mode 1, the response is mainly concentrated in a single displacement component, while in Mode 2, both displacement components show synchronous differences in response to mass changes. Therefore, Mode 2 is more conducive to improving the accuracy of mass estimation.
4. The dual-mode mechanism provides more information sources for mass sensing: Mode 1 provides univariate response characteristics in the flat state, while Mode 2 provides multivariate response characteristics in the buckled state. The mass and position can be estimated by utilizing the different changes in the Hamiltonian function and displacement under the two modes.
5. The results further indicate that the vibration frequency and displacement amplitude can serve as practical readout quantities for mass sensing. The added mass changes the equivalent inertia of the system, and this effect is reflected in measurable variations in frequency and amplitude. When the added-mass location or magnitude changes, the two vibration modes exhibit different amplitude and frequency responses. Therefore, by jointly using the frequency and amplitude information from Mode 1 and Mode 2, the added-mass magnitude and attachment position can be more accurately identified.

## Data Availability

The original contributions presented in this study are included in the article. Further inquiries can be directed to the corresponding author.

## Appendix A

### Appendix A.1

The coefficient derivations for Equations (5) and (11) in Mode 1 are presented as follows:

(A1) $$\kappa =\frac{\partial^{2}u_{1}}{\partial z^{2}}=-\frac{4\pi^{2}}{L^{2}}\cos\left(\frac{2\pi z}{L}\right)\Delta a$$

(A2) $$\lambda -1=\frac{1}{2}{\left(\frac{\partial u_{1}}{\partial z}\right)}^{2}+\frac{\partial u_{3}}{\partial z}=\frac{2\pi^{2}}{L^{2}}\sin^{2}\left(\frac{2\pi z}{L}\right)\Delta a^{2}$$

(A3) $$\begin{array}{cc} W_{m} & =\frac{1}{2}\int_{-L/2}^{L/2}E_{Base}bh_{Base}\frac{4\pi^{4}}{L^{4}}\sin^{4}\left(\frac{2\pi z}{L}\right)\Delta a^{4}dz+\frac{1}{2}\int_{-L_{Film}/2}^{L_{Film}/2}E_{Film}bh_{Film}\frac{4\pi^{4}}{L^{4}}\sin^{4}\left(\frac{2\pi z}{L}\right)\Delta a^{4}dz\\ & =\frac{3\pi^{4}E_{Base}bh_{Base}}{4L^{3}}\Delta a^{4}\\ & +E_{Film}bh_{Film}\left[\frac{3\pi^{4}L_{Film}}{4L^{4}}-\frac{\pi^{3}}{2L^{3}}\sin\left(\frac{2\pi L_{Film}}{L}\right)+\frac{\pi^{3}}{16L^{3}}\sin\left(\frac{4\pi L_{Film}}{L}\right)\right]\Delta a^{4} \end{array}$$

(A4) $$\begin{array}{cc} W_{b} & =\frac{1}{24}\int_{-L/2}^{L/2}E_{Base}bh_{Base}^{3}\frac{16\pi^{4}}{L^{4}}\cos^{2}\left(\frac{2\pi z}{L}\right)\Delta a^{2}dz\\ & +\frac{1}{8}\int_{-L_{Film}/2}^{L_{Film}/2}E_{Film}bh_{Film}h_{Base}^{2}\frac{16\pi^{4}}{L^{4}}\cos^{2}\left(\frac{2\pi z}{L}\right)\Delta a^{2}dz\\ & ==\frac{\pi^{4}E_{Base}bh_{Base}^{3}}{3L^{3}}\Delta a^{2}\\ & +E_{Film}bh_{Film}h_{Base}^{2}\left[\frac{\pi^{4}L_{Film}}{L^{4}}+\frac{\pi^{3}}{2L^{3}}\sin\left(\frac{2\pi L_{Film}}{L}\right)\right]\Delta a^{2} \end{array}$$

(A5) $$\frac{\partial u_{1}}{\partial t}=\left[1+\cos\left(\frac{2\pi z}{L}\right)\right]\Delta \dot{a}$$

(A6) $$\frac{\partial u_{3}}{\partial t}=0$$

(A7) $$\begin{array}{cc} T_{1}(\Delta \dot{a}) & =\frac{1}{2}\int_{-L/2}^{L/2}\rho_{\mathrm{Base}}bh_{\mathrm{Base}}\left[1+2\cos\left(\frac{2\pi z}{L}\right)+\cos^{2}\left(\frac{2\pi z}{L}\right)\right]\Delta {\dot{a}}^{2}\mathrm{dz}\\ & +\frac{1}{2}\int_{-LFilm/2}^{LFilm/2}\rho_{\mathrm{Film}}bh_{\mathrm{Film}}\left[1+2\cos\left(\frac{2\pi z}{L}\right)+\cos^{2}\left(\frac{2\pi z}{L}\right)\right]\Delta {\dot{a}}^{2}\mathrm{dz}\\ & =(m\rho_{\mathrm{Base}}bh_{\mathrm{Base}}L+m_{Film}\rho_{Film}bh_{Film}L)\Delta {\dot{a}}^{2}\\ & =\frac{3}{4}\rho_{\mathrm{Base}}bh_{\mathrm{Base}}L\Delta {\dot{a}}^{2}+\left[\frac{3L_{Film}}{4}+\frac{L}{\pi }\sin\left(\frac{\pi L_{Film}}{L}\right)+\frac{L}{8\pi }\sin\left(\frac{2\pi L_{Film}}{L}\right)\right]\rho_{Film}bh_{Film}L\Delta {\dot{a}}^{2} \end{array}$$

### Appendix A.2

The coefficient derivations for Equations (5) and (11) in Mode 2 are presented as follows:

(A8) $$\kappa =\frac{\partial^{2}u_{1}}{\partial z^{2}}=\frac{16\pi^{2}\Delta a}{L^{2}}\cos\left(\frac{4\pi z}{L}\right)-\frac{4\pi^{2}A}{L^{2}}\cos\left(\frac{2\pi z}{L}\right)$$

(A9) $$\lambda -1=\frac{1}{2}{\left(\frac{\partial u_{1}}{\partial z}\right)}^{2}+\frac{\partial u_{3}}{\partial z}=-\varepsilon_{compre}+\frac{\pi^{2}A^{2}}{L^{2}}\left[1-\cos\left(\frac{4\pi z}{L}\right)\right]+\frac{4\pi^{2}\Delta a^{2}}{L^{2}}\left[1-\cos\left(\frac{8\pi z}{L}\right)\right]$$

(A10) $$\begin{array}{cc} W_{m} & =\frac{1}{2}{\int_{-L/2}^{L/2}E_{Base}bh_{Base}\left\{-\varepsilon_{compre}+\frac{\pi^{2}A^{2}}{L^{2}}\left[1-\cos\left(\frac{4\pi z}{L}\right)\right]+\frac{4\pi^{2}\Delta a^{2}}{L^{2}}\left[1-\cos\left(\frac{8\pi z}{L}\right)\right]\right\}}^{2}dz\\ & +\frac{1}{2}\int_{-L_{Film}/2}^{L_{Film}/2}E_{Film}bh_{Film}{\left\{-\varepsilon_{compre}+\frac{\pi^{2}A^{2}}{L^{2}}\left[1-\cos\left(\frac{4\pi z}{L}\right)\right]+\frac{4\pi^{2}\Delta a^{2}}{L^{2}}\left[1-\cos\left(\frac{8\pi z}{L}\right)\right]\right\}}^{2}dz\\ & =E_{Base}bh_{Base}\left[\frac{L}{2}{\left(-\varepsilon_{compre}+\frac{\pi^{2}A^{2}}{L^{2}}\right)}^{2}+\frac{L}{4}{\left(\frac{\pi^{2}A^{2}}{L^{2}}\right)}^{2}+\frac{4\pi^{2}}{L}\left(-\varepsilon_{compre}+\frac{\pi^{2}A^{2}}{L^{2}}\right)\Delta a^{2}+\frac{12\pi^{4}}{L^{3}}\Delta a^{4}\right]\\ & +E_{Film}bh_{Film}\left\{\begin{array}{l} \frac{L_{Film}}{2}{\left(-\varepsilon_{compre}+\frac{\pi^{2}A^{2}}{L^{2}}\right)}^{2}-\frac{L}{2\pi }{\left(-\varepsilon_{compre}+\frac{\pi^{2}A^{2}}{L^{2}}\right)}^{2}\left(\frac{\pi^{2}A^{2}}{L^{2}}\right)\sin\left(\frac{2\pi L_{Film}}{L}\right)\\ +{\left(\frac{\pi^{2}A^{2}}{L^{2}}\right)}^{2}\left[\frac{L_{Film}}{4}+\frac{L}{16\pi }\sin\left(\frac{4\pi L_{Film}}{L}\right)\right]\\ +\frac{4\pi^{2}\Delta a^{2}}{L^{2}}\left[\begin{array}{l} L_{Film}\left(-\varepsilon_{compre}+\frac{\pi^{2}A^{2}}{L^{2}}\right)-\frac{L}{4\pi }\left(\frac{\pi^{2}A^{2}}{L^{2}}\right)\sin\left(\frac{2\pi L_{Film}}{L}\right)\\ -\frac{L}{4\pi }\left(-\varepsilon_{compre}+\frac{\pi^{2}A^{2}}{L^{2}}\right)\sin\left(\frac{4\pi L_{Film}}{L}\right)\\ +\frac{L}{12\pi }\left(\frac{\pi^{2}A^{2}}{L^{2}}\right)\sin\left(\frac{6\pi L_{Film}}{L}\right) \end{array}\right]\\ +\Delta a^{4}\left[\frac{12\pi^{4}L_{Film}}{L^{4}}-\frac{4\pi^{3}}{L^{3}}\sin\left(\frac{4\pi L_{Film}}{L}\right)+\frac{\pi^{3}}{2L^{3}}\sin\left(\frac{8\pi L_{Film}}{L}\right)\right] \end{array}\right\} \end{array}$$

(A11) $$\begin{array}{cc} W_{b} & =\frac{1}{24}\int_{-L/2}^{L/2}E_{Base}bh_{Base}^{3}\frac{16\pi^{4}}{L^{4}}\left[\begin{array}{l} A^{2}\cos^{2}\left(\frac{2\pi z}{L}\right)-8A\Delta a\cos\left(\frac{2\pi z}{L}\right)\cos\left(\frac{4\pi z}{L}\right)\\ +16\Delta a^{2}\cos^{2}\left(\frac{4\pi z}{L}\right) \end{array}\right]dz\\ & +\frac{1}{8}\int_{-L_{Film}/2}^{L_{Film}/2}E_{Film}bh_{Film}h_{Base}^{2}\frac{16\pi^{4}}{L^{4}}\left[\begin{array}{l} A^{2}\cos^{2}\left(\frac{2\pi z}{L}\right)-8A\Delta a\cos\left(\frac{2\pi z}{L}\right)\cos\left(\frac{4\pi z}{L}\right)\\ +16\Delta a^{2}\cos^{2}\left(\frac{4\pi z}{L}\right) \end{array}\right]dz\\ & =E_{Base}bh_{Base}^{3}\left[\frac{\pi^{4}A^{2}L_{Film}}{L^{4}}+\frac{16\pi^{4}}{3L^{3}}\Delta a^{2}\right]\\ & +E_{Film}bh_{Film}h_{Base}^{2}\left[\begin{array}{l} \frac{\pi^{4}A^{2}L_{Film}}{L^{4}}+\frac{\pi^{3}A^{2}}{2L^{3}}\sin\left(\frac{2\pi L_{Film}}{L}\right)-\frac{8\pi^{3}A}{L^{3}}\sin\left(\frac{\pi L_{Film}}{L}\right)\Delta a\\ -\frac{8\pi^{3}A}{3L^{3}}\sin\left(\frac{3\pi L_{Film}}{L}\right)\Delta a+\frac{16\pi^{4}L_{Film}}{L^{4}}\Delta a^{2}+\frac{4\pi^{3}}{L^{3}}\sin\left(\frac{4\pi L_{Film}}{L}\right)\Delta a^{2} \end{array}\right] \end{array}$$

(A12) $$\frac{\partial u_{1}}{\partial t}=\left[1-\cos\left(\frac{4\pi z}{L}\right)\right]\Delta \dot{a}$$

(A13) $$\frac{\partial u_{3}}{\partial t}=\Delta \dot{a}\left(\frac{2\pi A}{L}\sin\left(\frac{2\pi z}{L}\right)-\frac{2\pi A}{3L}\sin\left(\frac{6\pi z}{L}\right)\right)$$

(A14) $$\begin{array}{cc} T_{1}(\Delta \dot{a}) & =2\int_{-L/2}^{L/2}\rho_{\mathrm{Base}}bh_{\mathrm{Base}}\sin^{4}\left(\frac{2\pi z}{L}\right)\left[1+\frac{16\pi^{2}A^{2}}{9L^{2}}\sin^{2}\left(\frac{2\pi z}{L}\right)\right]\Delta {\dot{a}}^{2}\mathrm{dz}\\ & +2\int_{-LFilm/2}^{LFilm/2}\rho_{\mathrm{Film}}bh_{\mathrm{Film}}\sin^{4}\left(\frac{2\pi z}{L}\right)\left[1+\frac{16\pi^{2}A^{2}}{9L^{2}}\sin^{2}\left(\frac{2\pi z}{L}\right)\right]\Delta {\dot{a}}^{2}\mathrm{dz}\\ & =\Delta a^{2}\rho_{\mathrm{Base}}bh_{\mathrm{Base}}\left(\frac{3}{4}+\frac{10}{9}\varepsilon_{compre}-\frac{10\pi^{2}h_{Base}^{2}}{27L^{2}}\right)\\ & +\Delta a^{2}\rho_{\mathrm{Film}}bh_{\mathrm{Film}}\left\{\begin{array}{c} \frac{3L_{Film}}{8L}-\frac{5\pi^{2}h_{Base}^{2}L_{Film}}{27L^{3}}-\frac{5}{8\pi }\sin\left(\frac{\pi L_{Film}}{L}\right)\cos\left(\frac{\pi L_{Film}}{L}\right)\\ +\frac{1}{4\pi }\sin\left(\frac{\pi L_{Film}}{L}\right)\cos^{3}\left(\frac{\pi L_{Film}}{L}\right)\\ +\frac{\pi h_{Base}^{2}\sin\left(\frac{\pi L_{Film}}{L}\right)\cos\left(\frac{\pi L_{Film}}{L}\right)}{L^{2}}\left[\frac{11}{27}-\frac{26}{81}\cos^{2}\left(\frac{\pi L_{Film}}{L}\right)+\frac{8}{81}\sin^{4}\left(\frac{\pi L_{Film}}{L}\right)\right]\\ +\left[\begin{array}{l} \frac{5L_{Film}}{9L}-\frac{11}{9\pi }\sin\left(\frac{\pi L_{Film}}{L}\right)\cos\left(\frac{\pi L_{Film}}{L}\right)\\ +\frac{26}{27\pi }\sin\left(\frac{\pi L_{Film}}{L}\right)\cos^{3}\left(\frac{\pi L_{Film}}{L}\right)-\frac{8}{27\pi }\sin\left(\frac{\pi L_{Film}}{L}\right)\cos^{5}\left(\frac{\pi L_{Film}}{L}\right) \end{array}\right]\varepsilon_{compre} \end{array}\right\} \end{array}$$

### Appendix A.3

The resulting coefficients m, $m_{Film}$, $k_{i}$ and $k_{i,Film}$ $\left(i=0,1,2,3\right)$ of Mode 1:

(A15) $$\left\{\begin{array}{c} k_{0}=0,\\ k_{1}=0,\\ k_{2}=\frac{\pi^{4}}{3},\\ k_{3}=\frac{3\pi^{4}}{4h_{Base}^{2}}. \end{array}\right.$$

(A16) $$\left\{\begin{array}{c} k_{0,Film}=0,\\ k_{1,Film}=0,\\ k_{2,Film}=\frac{L_{Film}\pi^{4}}{L}+\frac{\pi^{3}}{2}\sin\left(\frac{2\pi L_{Film}}{L}\right),\\ k_{3,Film}=\frac{3\pi^{4}L_{Film}}{4Lh_{Base}^{2}}+\frac{\pi^{3}}{16h_{Base}^{2}}\sin\left(\frac{4\pi L_{Film}}{L}\right)-\frac{\pi^{3}}{2h_{Base}^{2}}\sin\left(\frac{2\pi L_{Film}}{L}\right). \end{array}\right.$$

(A17) $$m=\frac{3}{4}$$

(A18) $$m_{Film}=\frac{3L_{Film}}{4}+\frac{L}{\pi }\sin\left(\frac{\pi L_{Film}}{L}\right)+\frac{L}{8\pi }\sin\left(\frac{2\pi L_{Film}}{L}\right)$$

### Appendix A.4

The resulting coefficients m, $m_{Film}$, $k_{i}$ and $k_{i,Film}$ $\left(i=0,1,2,3\right)$ of Mode 2:

(A19) $$\left\{\begin{array}{c} k_{0}=\frac{\pi^{2}L^{2}\varepsilon_{compre}}{3}-\frac{\pi^{4}h_{Base}^{2}}{18},\\ k_{1}=0,\\ k_{2}=4\pi^{4},\\ k_{3}=\frac{12\pi^{4}}{h_{Base}^{2}}. \end{array}\right.$$

(A20) $$\left\{\begin{array}{cc} k_{0,Film} & =\frac{\pi^{2}LL_{Film}\varepsilon_{compre}}{2}-\frac{5\pi^{4}L_{Film}h_{Base}^{2}}{36L}+\frac{\pi L^{2}\varepsilon_{compre}}{2}\sin\left(\frac{\pi L_{Film}}{L}\right)\cos\left(\frac{\pi L_{Film}}{L}\right)\\ & -\frac{\pi^{3}h_{Base}^{2}}{6}\sin\left(\frac{\pi L_{Film}}{L}\right)\cos\left(\frac{\pi L_{Film}}{L}\right)\\ k_{1,\mathrm{Film}} & =\frac{16\pi^{2}L}{3}\sin^{3}\left(\frac{\pi L_{Film}}{L}\right)\sqrt{\varepsilon_{compre}-\frac{\pi^{2}h^{2}}{3L^{2}}}-8\pi^{2}L\sin\left(\frac{\pi L_{Film}}{L}\right)\sqrt{\varepsilon_{compre}-\frac{\pi^{2}h^{2}}{3L^{2}}}\\ k_{2,\mathrm{Film}} & =\frac{22\pi^{4}L_{Film}}{3L}+\frac{52\pi^{3}}{3}\cos^{3}\left(\frac{\pi L_{Film}}{L}\right)\sin\left(\frac{\pi L_{Film}}{L}\right)-\frac{26\pi^{3}}{3}\sin\left(\frac{\pi L_{Film}}{L}\right)\cos\left(\frac{\pi L_{Film}}{L}\right)\\ k_{3,Film} & =\frac{6\pi^{4}L_{Film}}{h_{Base}^{2}L}-\frac{16\pi^{3}}{h_{Base}^{2}}\sin\left(\frac{\pi L_{Film}}{L}\right)\cos^{3}\left(\frac{\pi L_{Film}}{L}\right)\\ & +\frac{10\pi^{3}}{h_{Base}^{2}}\sin\left(\frac{\pi L_{Film}}{L}\right)\cos\left(\frac{\pi L_{Film}}{L}\right)-\frac{4\pi^{3}}{h_{Base}^{2}}\sin^{3}\left(\frac{\pi L_{Film}}{L}\right)\cos\left(\frac{\pi L_{Film}}{L}\right)\\ & -\frac{16\pi^{3}}{h_{Base}^{2}}\sin^{3}\left(\frac{\pi L_{Film}}{L}\right)\cos^{3}\left(\frac{\pi L_{Film}}{L}\right)+\frac{32\pi^{3}}{h_{Base}^{2}}\sin^{5}\left(\frac{\pi L_{Film}}{L}\right)\cos^{3}\left(\frac{\pi L_{Film}}{L}\right) \end{array}\right.$$

(A21) $$m=\frac{3}{4}+\frac{10}{9}\varepsilon_{compre}-\frac{10\pi^{2}h_{Base}^{2}}{27L^{2}}$$

(A22) $$\begin{array}{cc} m_{Film} & =\frac{3L_{Film}}{8L}-\frac{5\pi^{2}h_{Base}^{2}L_{Film}}{27L^{3}}-\frac{5}{8\pi }\sin\left(\frac{\pi L_{Film}}{L}\right)\cos\left(\frac{\pi L_{Film}}{L}\right)\\ & +\frac{1}{4\pi }\sin\left(\frac{\pi L_{Film}}{L}\right)\cos^{3}\left(\frac{\pi L_{Film}}{L}\right)\\ & +\frac{\pi h_{Base}^{2}\sin\left(\frac{\pi L_{Film}}{L}\right)\cos\left(\frac{\pi L_{Film}}{L}\right)}{L^{2}}\left[\frac{11}{27}-\frac{26}{81}\cos^{2}\left(\frac{\pi L_{Film}}{L}\right)+\frac{8}{81}\sin^{4}\left(\frac{\pi L_{Film}}{L}\right)\right]\\ & +\left[\begin{array}{l} \frac{5L_{Film}}{9L}-\frac{11}{9\pi }\sin\left(\frac{\pi L_{Film}}{L}\right)\cos\left(\frac{\pi L_{Film}}{L}\right)\\ +\frac{26}{27\pi }\sin\left(\frac{\pi L_{Film}}{L}\right)\cos^{3}\left(\frac{\pi L_{Film}}{L}\right)-\frac{8}{27\pi }\sin\left(\frac{\pi L_{Film}}{L}\right)\cos^{5}\left(\frac{\pi L_{Film}}{L}\right) \end{array}\right]\varepsilon_{compre} \end{array}$$

### Appendix A.5

(A23)
$$\Delta \ddot{a}(t)=-A_{1}\omega^{2}\cos\left(\omega t\right)$$

(A24)
$$\Delta a^{3}(t)=A_{0}^{3}+3A_{0}^{2}A_{1}\cos\left(\omega t\right)+3A_{0}A_{1}^{2}\cos^{2}\left(\omega t\right)+A_{1}^{3}\cos^{3}\left(\omega t\right)$$

(A25)
$$\cos^{2}\left(\omega t\right)=\frac{1+\cos\left(2\omega t\right)}{2}$$

(A26)
$$\cos^{3}\left(\omega t\right)=\frac{3\cos\left(\omega t\right)+\cos\left(3\omega t\right)}{4}$$

Ignoring higher harmonics and setting the coefficients to zero, we can obtain the following by balancing the constant term:

(A27) $$\left(K_{1}+K_{1,Film}\right)+2\left(K_{2}+K_{2,Film}\right)A_{0}+6\left(K_{3}+K_{3,Film}\right)A_{1}^{2}A_{0}+4\left(K_{3}+K_{3,Film}\right)A_{0}^{3}=0$$

By balancing the fundamental frequency term, we can obtain

(A28) $$M\omega^{2}=2\left(K_{2}+K_{2,Film}\right)+12\left(K_{3}+K_{3,Film}\right)A_{0}^{2}+3\left(K_{3}+K_{3,Film}\right)A_{1}^{2}$$
